# Engineering the A-site cations in (Na/K/Rb)_3_InBr_6_ halides for enhanced optoelectronic and thermoelectric performance in renewable energy devices

**DOI:** 10.1039/d6ra01760f

**Published:** 2026-04-17

**Authors:** Hudabia Murtaza, Mohamed A. Habib, Quratul Ain, Ahmed B. M. Ibrahim, Junaid Munir

**Affiliations:** a Department of Physics, University of Management and Technology Lahore Pakistan; b Department of Chemistry, College of Science, Imam Mohammad Ibn Saud Islamic University (IMSIU) Riyadh 11623 Saudi Arabia; c Department of Physics, Riphah International University Lahore Pakistan junaid_ij2000@yahoo.com

## Abstract

Perovskite halides are a subclass of perovskite materials that are widely known for their exceptional compositional tunability and structural flexibility. This manuscript reports first principles analysis of the (Na/K/Rb)_3_InBr_6_ perovskite halides. The structural analysis for all three halides is validated based on the optimization curves and formation energies. The obtained tolerance factors and octahedral titling indicate minimum distortions in the formation of the cubic structure of (Na/K/Rb)_3_InBr_6_. The stress-energy tensor matrix is employed to assess the elastic constants of A_3_InBr_6_ (A = Na, K, and Rb). The transverse rigidity, longitudinal stiffness and ductility decline when the A-site cation is exchanged from Na to Rb. The elastic anisotropy computed using the ELATE software indicates that among the investigated compounds, K_3_InBr_6_ displays comparatively reduced anisotropy and a more uniform elastic response, but Na_3_InBr_6_ and Rb_3_InBr_6_ show pronounced directional changes. This highlights the function of the alkali-ion size in modifying the mechanical properties of these halide systems. Direct bandgaps of 3.37 eV, 3.84 eV and 3.86 eV were determined for Na_3_InBr_6,_ K_3_InBr_6_ and Rb_3_InBr_6_, respectively. The partial density of states plots indicate that for all studied halides, the Br-p states have a high contribution to the valence band. Optical analysis of Na_3_InBr_6,_ K_3_InBr_6_ and Rb_3_InBr_6_ reveals a prominent peak and electronic excitations in the UV region. The Seebeck coefficient of the studied halides is high at low temperatures, while the electrical and thermal conductivities increase with temperature. The *ZT* values suggest that Na_3_InBr_6,_ K_3_InBr_6_ and Rb_3_InBr_6_ are strong candidates for thermoelectric devices.

## Abstract 2

This research article provide the electronic, structural, mechanical, hydrogen storage and optical of (Na/K/Rb)_3_InBr_6_ using density functional theory implemented in wien2k code. The mBJ potential is used to solve the exchange–correlation potential.

## Graphical abstract

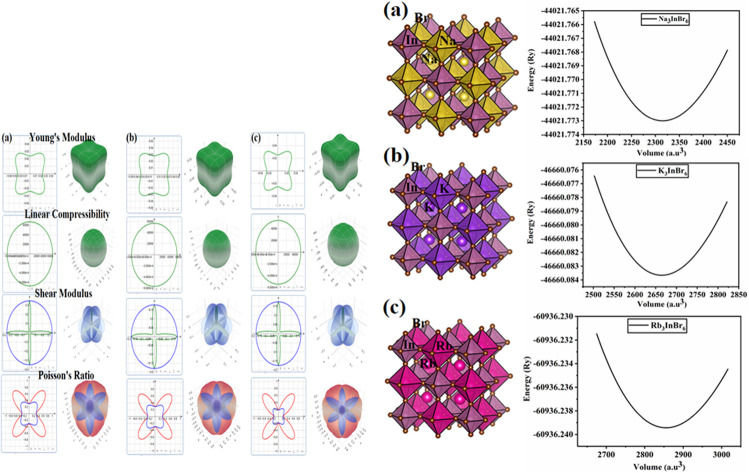

## Introduction

Renewable energy sources are being developed as feasible and ecologically benign options for meeting the energy needs of the world. Global population growth and industrialization have resulted in a huge increase in energy demand.^1^ In this technological age, the world faces numerous problems, one of which is the attainment of clean and affordable energy, which has to be addressed as soon as possible.^2^ The intensive use of fossil fuels contributes significantly to carbon emissions, which are harmful for humans and all living beings.^3,4^ The negative impact of the intensive use of fossil fuels as an energy source has disrupted the climate equilibrium.^5^ The world is experiencing extreme weather occurrences as a result of global warming and human activity. The only way to attain zero carbon and fulfill the uninterrupted energy supply is to search for materials that are cost-effective and environmentally benign.^6–8^ It is now a top priority of researchers to develop renewable energy solutions as the consumption of large amounts of energy generated from fossil fuels is creating environmental problems.^9–11^ The search for unique materials with specified qualities is critical for advancing research and technology within materials research.^12–14^ Solar energy, in particular, is an important and long-term option that has lately been identified as the most cost-effective method of electricity generation.^15,16^ Understanding the technical applications, such as deep-UV nonlinear optical responses, is vital for evaluating material performance and inherent qualities.^17^ An extraordinary class of materials, known as perovskite materials, has been in demand due their wide range of applications and structural flexibility.^18,19^ In recent years, there has been an explosion of research on perovskite materials in photovoltaics and photoluminescence.^20,21^ Furthermore, halide perovskites with alkali metal ions possess the tendency to enhance the material brightness, bandgap and moisture resistance.^22–24^ Multiple perovskite halides have been investigated in the literature, such as lead-free perovskites Cs_3_Ag_2_X_5_ (X = I, Br, and Cl), by analyzing the GGA and HSE06 potentials, demonstrating that these halides exhibit strong prominent peaks in the UV region.^25^ RbZnX_3_ (X = Br, Cl, and F) halides possess indirect bandgaps of 0.103 eV, 1.387 eV and 3.63 eV, respectively, making them promising candidates for photovoltaic applications.^26^ The halides AlSnX_3_ (X = F, Cl, Br and I) were analyzed using different potentials. Analysis of all optical properties reveal that the studied halides exhibit excellent optical responses in the visible region.^27^ Double perovskites Z_2_NaCrCl_6_ (Z = K and Rb) were investigated using first principles analysis. Both studied halides are thermodynamically and mechanically stable.^28^ The electronic analysis of K_2_TlZI_6_ (Z = Al and In) suggests that the materials are viable for photovoltaic applications.^29^ A computational method was employed to investigate the physical attributes of Cs_2_KAsA_6_ (A = Cl, Br, and I). The mechanical attributes suggest that both halides are ductile and are viable for renewable energy devices.^30^ Using SCAPS-1D simulations, the properties of X_2_SnBr_6_ revealed direct bandgaps and a power energy conversion efficiency up to 30.62%.^31^ Thermoelectric analysis of Na_2_Sn(Cl/Br)_6_ reveals that both halides possess favorable *ZT* values.^32^ The BoltzTraP code was used to assess the transport performance of K_2_ReX_6_. The studied halides exhibit high *ZT* values which make them effective thermoelectric materials.^33^ The FP-LAPW approach is used for studying the physical attributes of LiMgI_3_ and NaMgI_3_ halides. First principles investigation reveals that both halides are ductile and exhibit indirect bandgaps. Furthermore, the thermoelectric properties reveal favorable transport parameters at room temperature, making them potential materials for green energy.^34^ DFT analysis of A_2_AlInI_6_ halides indicates strong absorption in the visible region and promising photovoltaic efficiencies determined *via* SLME. For all halides, the *ZT* value is approximately 0.7, revealing their potential for solar–thermoelectric renewable energy applications.^35^ The physical properties of Cs_2_GaBiX_6_ halides were predicted using different functionals. Comprehensive analysis of these attributes suggests that these halides are optically and thermally stable.^36^ DFT simulations of A_2_AgRhF_6_ (A = Na and Rb) show that they are indirect semiconductors with strong absorption in the UV-vis region. Thermoelectric analysis of the reported halides exhibits high *ZT* values, which indicate strong potential thermoelectric applications.^37^ As a thermoelectric attribute, Li_2_ATlCl_6_ (A = Na and K) demonstrates high thermoelectric performance at 1200 K.^38^ The physical attributes of Z_2_BIrCl_6_ (Z = Cs and Rb and B = Na and K) were determined using the mBJ approximation. The obtained bandgaps suggest that the material possesses strong absorption in the visible range.^39^ The CASTEP code was used to evaluate the physical attributes of AgSrM_3_. Evaluation of the mechanical properties indicates that the studied halides are ductile.^40^ The phonon curves of Cs_2_SiCl_6_, Cs_2_GeCl_6_, and Cs_2_SnCl_6_ indicate the structural stability of these halides.^41^ The present study introduces (Na/K/Rb)_3_InBr_6_ halides as a previously unexplored class of indium-based bromide perovskite-related materials, which are investigated *via* first principles analysis. This work provides the first systematic comparison of alkali-metal substitution at the A-site and its impact on structural stability and lattice distortions. The study reveals composition-dependent electronic bandgap tunability, which allows for controlled regulation of the optoelectronic properties. Comprehensive mechanical investigation verifies the elastic stability and ductile behavior, which have not been described before for this family. The optical response exhibits high visible-light absorption and low reflectance, indicating their potential for optoelectronic devices. These findings identify (Na/K/Rb)_3_InBr_6_ halides as innovative, stable, and adjustable materials for optoelectronic and energy-related applications.

### Computational approach

Unlike traditional wave-function-based quantum mechanical approaches, which are computationally prohibitively expensive for complicated materials, DFT reduces the many-body problem of interacting electrons and nuclei to an effective single-particle formulation.^42^ This simplification is accomplished by the Kohn–Sham formalism, which allows for efficient and reliable evaluation of electronic structures. In this study, the Wien2K code is utilized to analyze the physical properties of (Na/K/Rb)_3_InBr_6_ halides. The Kohn–Sham equations are solved using the FP-LAPW method, and electron–ion interactions are better described using the mBJ potential.^43^ The halides are structurally optimized using the PBE-GGA potential. The elastic characteristics are determined by evaluating the three independent elastic constants using the second-order stress–strain relation. To achieve accurate self-consistent field convergence, we set the computational parameters to *G*_max_ = 14 (a.u.)^−1^, *L*_max_ = 12, and *K*_max_ × RMT = 7. To prevent charge leakage and muffin-tin sphere overlap, the cut-off energy of −8 Ry was adopted. The muffin-tin radii for Na, K, Rb, In and Br are set to 2.47, 2.41, 1.53, 1.51, and 1.78 (a.u.)^−1^, respectively. To generate well-converged electronic structures, a dense *k*-point mesh with 1300 points in the first Brillouin zone is used. The SCF convergence criterion was set at 0.1 mRy for the total energy and 0.0001*e* for the electronic charge. The VESTA software was used to visualize the crystal formations. Furthermore, the optical response of (Na/K/Rb)_3_InBr_6_, including the dispersion and polarizability, were investigated using the Kramers–Kronig relationships.

### Structural analysis and volume optimization

**Fig. 1:**
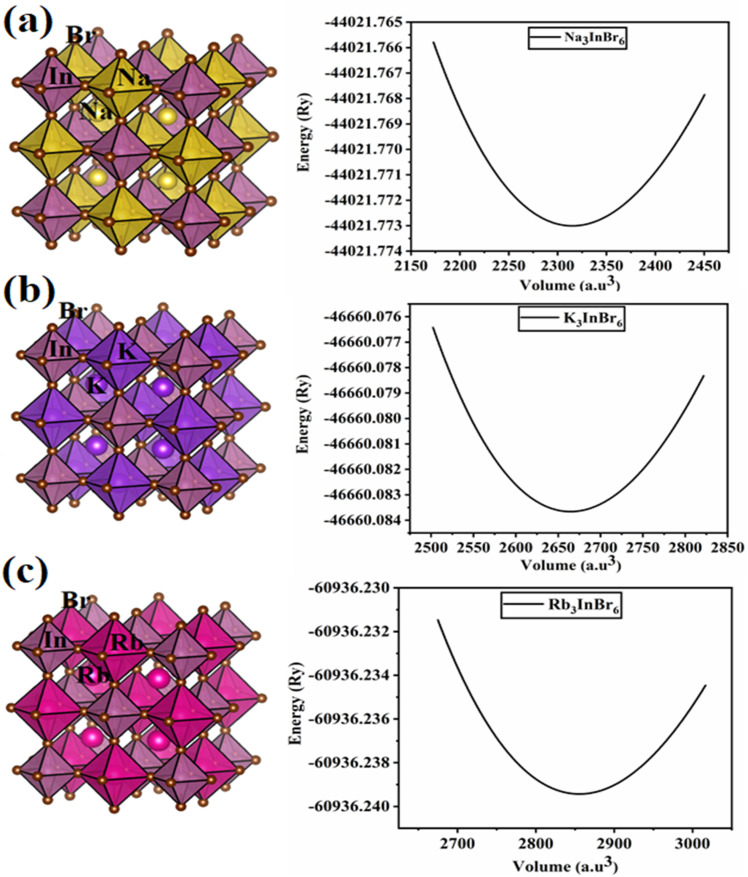
Unit cells and optimization curves of (a) Na_3_InBr_6_ (b) K_3_InBr_6_ and (c) Rb_3_InBr_6_.

Modifying the structure of a material can alter its physical attributes drastically. In halide perovskites, cation and anion replacement can alter the material's structural parameters which can affect its physical attributes. In this section, we discuss the structural parameters of (Na/K/Rb)_3_InBr_6_ halides. The studied halides possess the general formula of double perovskite, A_3_BX_6_, in which the A-site is occupied by sodium, potassium and rubidium, the B-site is occupied by indium and the X-site contains bromine. The structural properties of the studied halides (Na/K/Rb)_3_InBr_6_ are investigated using the VESTA software.^44^ The atomic positions are obtained as Na/K/Rb (1/4, ¼, ¼), K (0, 0, ½), In (0, 0, 0) and Br (0, 0, 0.254). The bond lengths for Na–Br, K–Br, Rb–Br and In–Br are given as 3.57715 Å, 3.8513 Å, 4.05498 Å and 3.05329 Å, respectively. The computed atomic bond lengths between the A-site cations and X-site halide increase when the A-site cation is changed from Na to K and from K to Rb. This increment in the bond length indicates lattice expansion and reduced coulombic interaction strength between the A-site cation (Na, K, Rb) and X-site anion (Br). Fig. 1(a–c) presents visual representations of the crystal structures of Na_3_InBr_6_, K_3_InBr_6_ and Rb_3_InBr_6_. The crystal structures were adjusted to achieve a stable geometry by reducing the total energy and atomic forces while maintaining structural stability.^45^ This optimization produces precise equilibrium lattice parameters and bond angles, all of which are required to properly predict the material's electrical, mechanical, and optical properties. The obtained optimization curves for Na_3_InBr_6_, K_3_InBr_6_ and Rb_3_InBr_6_ are presented in Fig. 1. The most stable configuration for the studied halides is obtained by fitting the optimization data into the following Birch–Murnaghan equation as follows:1
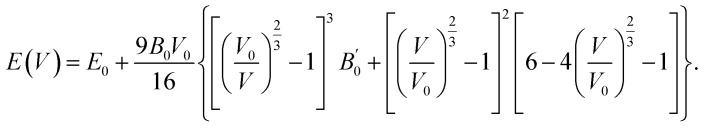


The obtained values from curve fitting for the studied halides are presented in Table 1. The obtained value of the lattice constant increases when the A-site cation is substituted as Na → K → Rb, related to expansion of the crystal structure. This suggests that further replacement of the heavier cation at the A-site in the studied halide can further increase the lattice constant of the studied halides. The reported bulk modulus suggests a decreasing trend in the materials resilience towards external strain when the A-site cation is replaced as Na → K → Rb. The negative energies at equilibrium volumes determined for Na_3_InBr_6,_ K_3_InBr_6_ and Rb_3_InBr_6_ suggest that the materials are structurally stable. The thermodynamic stability of the studied halides is assessed based on the formation energy, computed as follows:

2 *E*_f_ = *E*_(Na/K/Rb)_3_InBr_6__ − [3*E*_Na/K/Rb_ − *E*_In_ − 6*E*_Br_].

**Table 1:** Optimized structural attributes of (Na/K/Rb)_3_InBr_6_

Perovskites	Na_3_InBr_6_	K_3_InBr_6_	Rb_3_InBr_6_
*a* = *b* = *c* (Å)	11.11	11.645	11.917
Angle	90°		
Symmetry/space group	Cubic/*Fm*3̄*m*-225		
*B* (GPa)	22.49	18.96	18.079
*B*	5	5	5
*V*_0_ (a.u^3^)	2314.96	2664.43	2855.55
*E*_0_ (Ry)	−44021.773	−46660.083	−60936.23
*E*_F_ (Ry per atom)	−1.705	−1.815	−1.787
*τ*	0.88	0.86	0.88
*µ*	0.412	0.416	0.428

The negative formation energies of the material suggest that the material is thermodynamically stable. This suggests that the material is energetically favorable under equilibrium conditions.^46^ Here, *E*_(Na/K/Rb)_3_InBr_6__ is the ground-state energy, computed from the optimization curves; the individual energies for Na, K, Rb, In and Br are given as *E*_Na/K/Rb_, *E*_In_ and *E*_Br_. The computed formation energies for Na_3_InBr_6,_ K_3_InBr_6_ and Rb_3_InBr_6_ are negative, which suggests that these halides are energetically favorable under equilibrium conditions. The tolerance factor for the studied halides was also computed to assess the structural stability. For cubic materials, the tolerance factor is suggested to be in the range of 0.8–1.11.^47^ The octahedral tilting is a measure of the stability of BX_6_ octahedra, which lies in the range of 0.40–0.5 (ref. 48) for cubic materials with minimum distortions. The computed tolerance factor and octahedral tilting for Na_3_InBr_6,_ K_3_InBr_6_ and Rb_3_InBr_6_ suggest minimum distortions and complete structural stability.

### Phonon dispersion curves

**Fig. 2:**
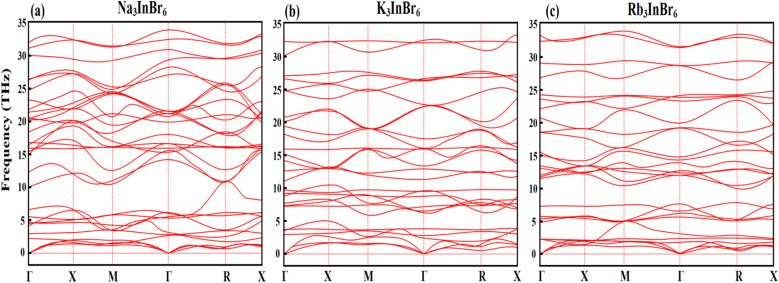
Phonon curves for determining the stability criteria of (a) Na_3_InBr_6_ (b) K_3_InBr_6_ and (c) Rb_3_InBr_6_.

Phonon dispersion curves are essential for assessing the dynamic stability of a material by identifying the presence or absence of imaginary frequencies. The phonon dispersion curves of Na_3_InBr_6_, K_3_InBr_6_, and Rb_3_InBr_6_ are plotted along the high-symmetry path (*Γ*–*X*–*M*–*Γ*–*R*–*X*) in Fig. 2. The curves demonstrate the dynamic stability of all three compounds, as no imaginary (negative) frequencies are observed across the Brillouin zone. Each system exhibits three acoustic branches originating from the *Γ*-point in the low-frequency region, corresponding to collective lattice vibrations, followed by multiple optical branches at higher frequencies due to the presence of multiple atoms in the unit cell. Additionally, the presence of moderately dispersive and partially flat optical modes suggests localized vibrations and enhanced phonon scattering, which can contribute to reduced lattice thermal conductivity. These features highlight the stability and potential suitability of these materials for thermoelectric and energy-related applications.

### Mechanical properties

The mechanical properties of a material describe the material's resistance and resilience to external deformation.^49^ A material's ability to expand and contract in the provided elastic limit can be determined by assessing its mechanical properties. The mechanical properties are analyzed using the elastic constant as a metric. These elastic constants are independent and are obtained according to the material's symmetry. The stress and strain of the material are proportional to each other within the provided elastic limit, which is defined by Hooke's law. The second-order strain-energy equation is written as a quadratic expansion of the strain tensor, which relates the elastic constants to the stored energy due to deformation.^50^ The elastic strain energy is described by the second-order strain-energy equation as a quadratic expansion of strain tensor components. In this approach, the elastic constants act as coefficients that link the applied strain to the energy stored during deformation. As a result, the equation establishes a basic relationship between the microscopic elastic constants and macroscopic mechanical response of a material to tiny, reversible deformations. The equation is given as follows:3
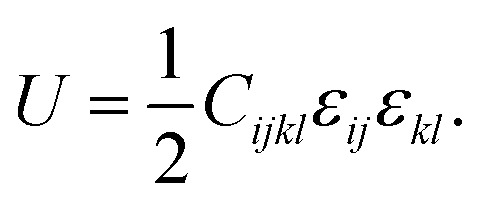


For the studied halides, three constants *C*_11_, *C*_12_ and *C*_44_ are obtained *via* the Thomas Charpin's technique.^45,47^ The calculated elastic constants for Na_3_InBr_6_, K_3_InBr_6_ and Rb_3_InBr_6_ fit Born's stability criteria. The elastic constant *C*_11_ indicates the material's ability to resist being stretched and compressed in one direction. *C*_12_ expresses how much the material's shape is altered in one direction when its pushed and pulled in another direction, whereas *C*_44_ defines how hard it is to slide one layer of the material over another.^47^ A higher *C*_11_ implies great resistance to uniaxial deformation, whereas lower *C*_44_ values imply moderate shear stiffness, which is good for structural flexibility and potential mechanical robustness under external stress. Among the studied halides, *C*_11_ for Na_3_InBr_6_ indicates the highest resistance to being stretched and compressed in one direction, as compared to K_3_InBr_6_ and Rb_3_InBr_6_. The value of *C*_12_ also indicates that Na_3_InBr_6_ possesses higher capacity for being pulled or pushed in another direction as compared to K_3_InBr_6_ and Rb_3_InBr_6_. Based on the computed value of *C*_44_, Rb_3_InBr_6_ demonstrates the highest level of shear deformation, as compared to K_3_InBr_6_ and Na_3_InBr_6_. The bulk modulus represents a material's capacity to be compressed when external pressure is applied.^51^ The bulk modulus for Na_3_InBr_6_ is moderately larger than that of K_3_InBr_6_ and Rb_3_InBr_6_, which suggests that more external pressure is required to compress Na_3_InBr_6_ than K_3_InBr_6_ and Rb_3_InBr_6_. The Na cation at the A-site of the material corresponds to stronger interatomic bonding and more compact crystal formation while remaining stable under high pressures. The toughness and stiffness of a material is indicated by the Young's modulus.^52^ The computed values of the Young's modulus suggest that Rb_3_InBr_6_ is stiffer and more rigid than Na_3_InBr_6_ and K_3_InBr_6_. This implies that an increase in the A-site cation ionic size can increase the material's stiffness. The shear modulus expresses the material's ability to resist shear deformation.^53^ When the A-site cation size is increased from Na to K and Rb, the computed shear modulus increases moderately, suggesting that Rb_3_InBr_6_ is more resistant to shear deformation, as compared with Na_3_InBr_6_ and K_3_InBr_6_. The anisotropy factor describes whether a material exhibits anisotropic or isotropic mechanical behavior. For a cubic system, an anisotropy factor of *A* = 1 indicates isotropic character, meaning the mechanical properties are independent of direction. Any deviation of *A* from 1 reflects anisotropic behavior.^54^ The anisotropy factor for the three halides indicates their anisotropic nature, indicating that the mechanical attributes are independent of the crystallographic directions. The Pugh's ratio is a common parameter indicating a material's ductile and brittle nature. Ductile materials are relatively tough and possess higher plastic deformation as compared to brittle materials, which are fragile and can easily break.^55,56^ This distinction results from the materials' differing atomic bonding and deformation mechanisms. Ductile materials can undergo extensive plastic deformation *via* dislocation motion prior to fracture, allowing them to absorb more energy under stress. Brittle materials, on the other hand, have restricted dislocation activity and fracture abruptly when the elastic limit is reached. As a result, brittle materials are often less appropriate for applications that require impact loading or mechanical stress fluctuations. The critical Pugh's ratio (*B*/*G*) for ductile materials should be greater than 1.75.^57^ The obtained values for the studied halides (Na_3_InBr_6,_ K_3_InBr_6_ and Rb_3_InBr_6_) indicates their ductile nature as the Pugh's ratio is higher than the critical value of 1.75. When the A-site is substituted from Na to K and Rb, the material's ductility declines as the ionic size increases. The Poisson's ratio is another parameter that further confirms the material's ductility and brittleness suggested by the computed Pugh's ratio.^56^ The computed Poisson's ratio suggests that Na_3_InBr_6_, K_3_InBr_6_ and Rb_3_InBr_6_ are all ductile, as their Poisson's ratio is higher than 0.26. The Cauchy's pressure is another term that also aids in assessing a material's ductility and brittleness. A positive Cauchy's pressure suggests ductile nature, whereas brittleness is indicated by negative values of Cauchy's pressure.^58^ The computed values of the Cauchy's pressure for Na_3_InBr_6_, K_3_InBr_6_ and Rb_3_InBr_6_ suggest that the studied halides are ductile, consistent with the Pugh's and Poisson's ratios. The hardness factor reflects the materials ability to resist localized plastic deformations such as scratching and abrasion.^59,60^ The obtained values of the hardness factor imply that Rb_3_InBr_6_ is more resistant to localized deformation as compared to K_3_InBr_6_ and Na_3_InBr_6_. This indicates that increasing the cation size at the A-site of the studied material can lead to higher resistance to localized plastic deformations such as scratching and abrasion. The machinability index indicates the level of ease through which the material can be manufactured or processed.^61^ The machinability index shows a declining trend, which suggests that as the A-site cation is replaced by larger ionic-size cations, its level of ease in manufacturing declines. Moreover, it is more convenient to manufacture Na_3_InBr_6_ as compared to K_3_InBr_6_ and Rb_3_InBr_6_. Elastic wave propagation provides a direct measure of the material's wave velocity. Furthermore, it indicates the material's stiffness and rigidity.^62^ The longitudinal velocity represents the speed of compressional elastic waves passing through the material. Larger longitudinal velocities suggest stronger interatomic bonding and greater stiffness. The computed longitudinal velocities reveal that K_3_InBr_6_ possesses stronger bonding and is stiffer than Na_3_InBr_6_ and Rb_3_InBr_6_. The transverse velocity reflects the speed of shear elastic waves and is directly related to the shear rigidity. Higher transverse velocities suggest that the material exhibits better resistance to shear deformation. The obtained transverse velocities suggest that Rb_3_InBr_6_ possesses higher resistance to shear deformation while Na_3_InBr_6_ possesses the lowest shear stability. The mean sound velocity combines both longitudinal and transverse wave contributions. It is an important parameter, which is crucial for assessing the thermal and vibrational properties of a material.^63^ The obtained values of the mean sound velocity indicate that Rb_3_InBr_6_ possesses stronger phonon propagation, whereas Na_3_InBr_6_ shows comparatively compliant lattice dynamics. The Debye temperature of a material describes the temperature required for activating the vibrational modes in the lattice. Moreover, stronger bonding and better thermal conductivity are indicated by a higher Debye temperature.^64^ From the obtained values for the studied halides, it is noticeable that K_3_InBr_6_ exhibits stronger atomic interactions and stiffer lattice vibrations. At the melting temperature, the crystal breaks down into the liquid phase, reflecting its thermal stability. High melting temperatures suggest that a material possesses better thermal robustness.^65,66^ The obtained melting temperature revealed that Na_3_InBr_6_ possesses better thermal stability than K_3_InBr_6_ and Rb_3_InBr_6_ (Table 2).

**Table 2:** Mechanical properties of Na_3_InBr_6_, K_3_InBr_6_ and Rb_3_InBr_6_

Parameters	Na_3_InBr_6_	K_3_InBr_6_	Rb_3_InBr_6_
*C*_11_ (GPa)	41.803	35.802	39.43
*C*_12_ (GPa)	12.81	10.52	7.365
*C*_44_ (GPa)	2.88	5.569	5.747
*B* (GPa)	22.49	18.96	18.079
*E* (GPa)	16.2	20.5	22.68
*S* (GPa)	5.88	7.77	8.79
*A*	0.19	0.43	0.35
*B*/*G*	3.82	2.43	2.05
*v*	0.37	0.31	0.29
*C*_p_ (GPa)	9.93	4.96	1.62
*H*_v_	0.512	0.99	1.23
*µ*_m_	7.80	3.41	3.14
*v*_l_ (m s^−1^)	4140.3	4197.28	4045.54
*v*_t_ (m s^−1^)	1823.75	2161.99	2198.33
*v*_m_ (m s^−1^)	2058.76	2420.92	2452.55
*θ*_D_ (K)	161.48	181.25	179.45
*T*_m_ (K)	800.056	764.58	786.03

### Elastic anisotropy

**Fig. 3:**
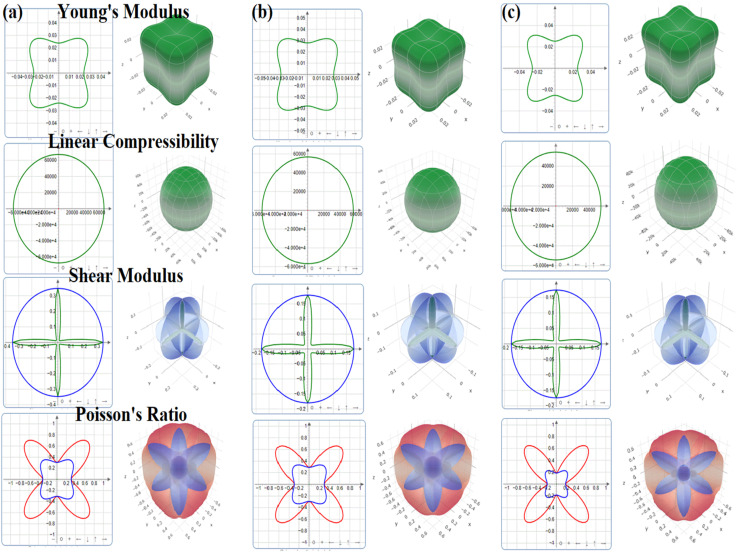
Elastic anisotropy plots of (a) Na_3_InBr_6_, (b) K_3_InBr_6_ and (c) Rb_3_InBr_6_.

Elastic anisotropy quantifies the difference in the mechanical characteristics across crystallographic orientations. An anisotropy of 1 suggests that a material is totally isotropic, whereas deviations imply directional dependence of the elasticity. The elastic anisotropy of the studied halides was further assessed by employing the ELATE software.^67^ This software generates 2D and 3D images of variation in the material's mechanical properties in the crystallographic direction, which helps in describing the bond strength and mechanical stability. Fig. 2 demonstrates the 2D and 3D visualization of the Young's modulus, linear compressibility, Shear modulus and Poisson's ratio for Na_3_InBr_6,_ K_3_InBr_6_ and Rb_3_InBr_6_. The Young's modulus quantifies the material's directional-dependent stiffness. A perfectly spherical shape indicates that a material is elastically isotropic, whereas lobed or non-spherical shapes indicate that a material is elastically anisotropic. Among the studied halides, K_3_InBr_6_ exhibits a smooth and more symmetric surface, suggesting lower anisotropy and uniform stiffness, whereas Na_3_InBr_6_ and Rb_3_InBr_6_ exhibit stronger anisotropy. The linear compressibility indicates the amount of compression a material can endure in one direction when external pressure is applied. The linear compressibility plots suggest that the obtained linear compressibility of all studied halides is less directionally dependent, as compared to the Young's modulus. Among the studied halides, Rb_3_InBr_6_ possesses enhanced linear compressibility compared to Na_3_InBr_6_ and K_3_InBr_6_. The shear modulus reflects the resistance to shape changes, which is critical for mechanical rigidity. The cross-like patterns in the 2D projections and the multi-lobed 3D surfaces show a considerable directional dependence. This anisotropy results from non-uniform resistance to shear deformation across distinct planes. Among the studied halides, K_3_InBr_6_ exhibits balanced shear behavior and Na_3_InBr_6_ and Rb_3_InBr_6_ display stronger anisotropy. Lastly, we assessed the transverse strain response to applied longitudinal stress, which is known as the Poisson's ratio. The very non-spherical, petal-like forms imply strong anisotropy in *v*. Directional variations indicate that bonding and angular forces vary greatly with orientation. For the studied halides, a variation in the ductility was noted in all crystallographic directions. In conclusion, all studied halides are elastically anisotropic despite being cubic. K_3_InBr_6_ exhibits the strongest isotropic mechanical response, suggesting better mechanical uniformity compared with Na_3_InBr_6_ and Rb_3_InBr_6_ (Fig. 3).

### Electronic properties

**Fig. 4:**
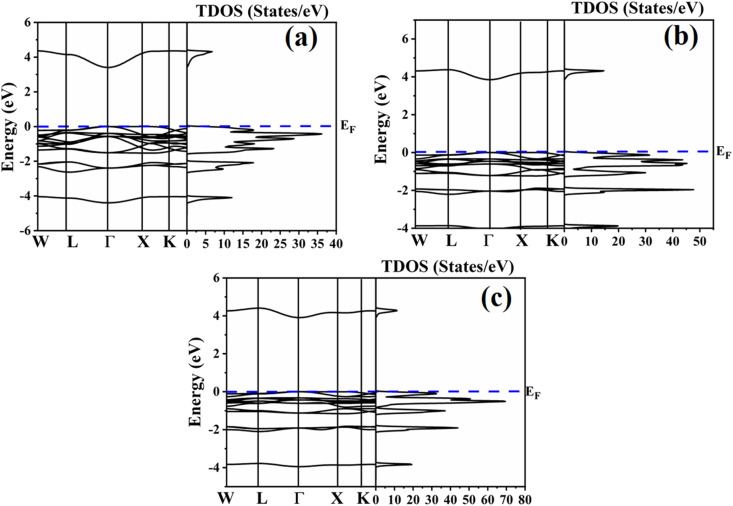
Electronic properties of (a) Na_3_InBr_6_ (b) K_3_InBr_6_ and (c) Rb_3_InBr_6_.

The nature of a material is determined by its electronic properties, and collectively, these properties govern the material's electronic behavior. Moreover, the electronic properties of the halides can be manipulated by altering the A-site cation or X-site anion, as these modifications alter the crystal structure and orbital hybridization in the material. The electronic properties of (Na/K/Rb)_3_InBr_6_ were obtained *via* the mBJ approximation and are presented in Fig. 4. All three studied halides exhibit no band-crossing at the Fermi level, indicating their semiconducting nature. A visible gap in the valence and conduction bands is visible for the studied halides, indicating no metallic behavior. The comparatively flat bands in both the valence and conduction regions indicate localized electronic states, which are typical of ionic halide perovskites. The obtained electronic bandgaps for Na_3_InBr_6,_ K_3_InBr_6_ and Rb_3_InBr_6_ are 3.37 eV, 3.84 eV and 3.86 eV, as depicted in Fig. 4(a–c). All studied halides have direct bandgaps. This systematic increase in the bandgap from Na_3_InBr_6_ to Rb_3_InBr_6_ is due to lattice expansion and reduced In–Br orbital hybridization. Furthermore, this change in the bandgap is also evident through the TDOS plots for all studied halides. The TDOS plots for Na_3_InBr_6,_ K_3_InBr_6_ and Rb_3_InBr_6_ also show a rising trend, which suggests that A-site substitution significantly modifies the electronic states by reducing orbital overlap, improving the ionic nature of the material, which leads to wide bandgaps. The band structure and TDOS investigations show that Na_3_InBr_6,_ K_3_InBr_6_ and Rb_3_InBr_6_ are wide-gap semiconductors with tunable electronic characteristics caused by A-site substitution. The ability to modify the bandgap by alkali-metal cation engineering makes these materials potential candidates for optoelectronic, insulating, and UV-related device applications that require chemical stability and band-gap control.

**Fig. 5:**
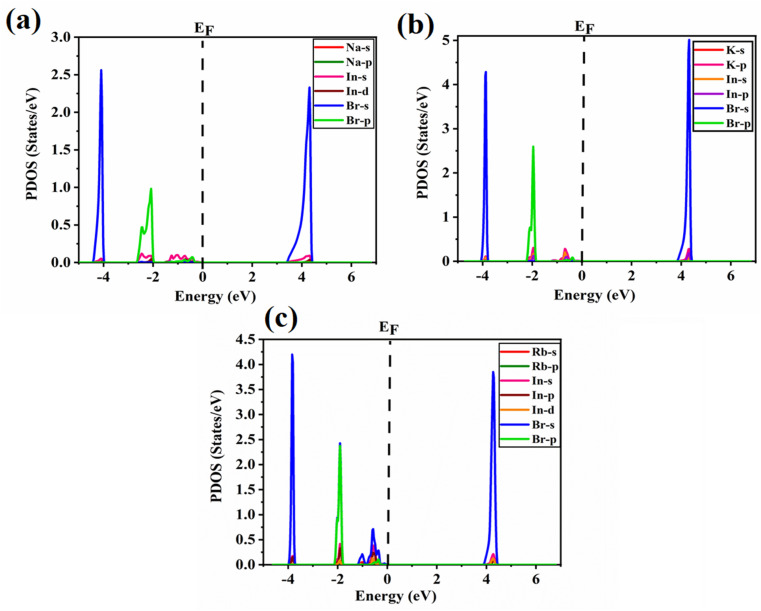
PDOS plots of (a) Na_3_InBr_6_, (b) K_3_InBr_6_ and (c) Rb_3_InBr_6_.

The partial density of states plots for Na_3_InBr_6,_ K_3_InBr_6_ and Rb_3_InBr_6_ are presented in Fig. 4(a–c). For Na_3_InBr_6_, the Na-s, Na-p, In-s and In-d orbitals have a minor contribution to the valence band, whereas a high contribution from the Br-s and Br-p states is observed. In the conduction band, the Br-s states are more prominent, along with a minor contribution from the In-s states, as depicted in Fig. 5(a). Fig. 5(b) demonstrates the partial density of states plot for K_3_InBr_6_, which indicates a minor contribution of the K-s, K-p, In-s and In-p orbitals to the valence band, whereas the majority contribution is from the Br-s and Br-p states. The conduction band has a high contribution from the Br-s states with a minor contribution from the K-p states. The valence band of Rb_3_InBr_6_ has a minor contribution from the Rb-s, Rb-p, In-s, In-p and In-d states, whereas the major contribution is from the Br-s and Br-p states. Furthermore, the conduction band has a large contribution from Br-s states, with minor contribution of In-s and In-d states, as depicted in Fig. 5(c).

### Optical properties

The material's interaction with light gives rise to multiple optical phenomena, which aids in predicting the material's suitability in various light-based devices.^68^ Optical analysis of the studied halides is performed using the complex Kramer–Kronig dielectric equations. The dielectric function exhibits real and the imaginary parts; it describes the material's dispersive behavior and optical absorption. The real and imaginary parts are expressed as follows:4
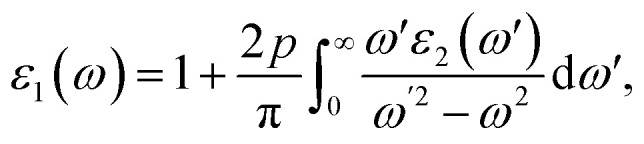
5
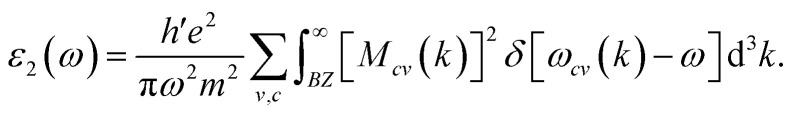


**Fig. 6:**
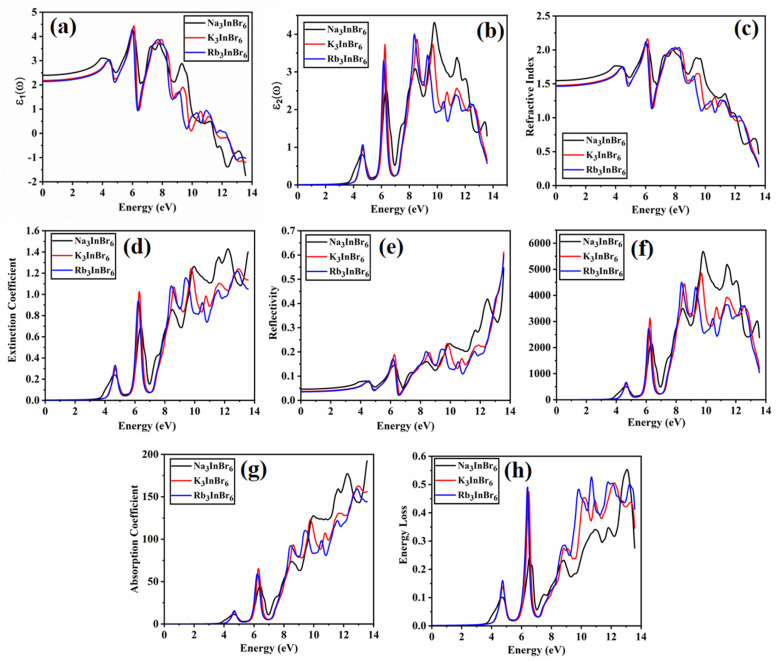
Optical properties of (Na/K/Rb)_3_InBr_6_.

Fig. 6 presents an optical analysis of Na_3_InBr_6_, K_3_InBr_6_ and Rb_3_InBr_6_. *ε*_1_(*ω*) for the studied halides is presented in Fig. 6(a), from which the static values are obtained as 2.39, 2.18 and 2.14 for Na_3_InBr_6_, K_3_InBr_6_ and Rb_3_InBr_6_, respectively. The decline in the static value is connected to the Penn's model in which the static value of the studied material is inversely related to the material's bandgap.^69^ With increasing energy, the *ε*_1_(*ω*) plot shows minor peaks in the UV region at 3.90 eV, 4.44 eV and 4.42 eV, whereas maximum peaks are observed at 6.02 eV, 6.10 eV and 6.02 eV for Na_3_InBr_6,_ K_3_InBr_6_ and Rb_3_InBr_6_, respectively. This means that the drop in the static dielectric constant is due to bandgap widening, which is compatible with Penn's concept, in which a bigger bandgap reduces the low-energy electronic polarizability. Minor peaks in the lower-UV region correlate to mild interband electronic transitions, but prominent maxima at higher UV energies imply substantial interband transitions from the valence to conduction states. These properties demonstrate that the optical response of Na_3_InBr_6,_ K_3_InBr_6_ and Rb_3_InBr_6_ is dominated by high-energy electronic excitations. The halides are optically active largely in the UV region and transparent in the visible range. *ε*_2_(*ω*) for the studied halides is presented in Fig. 6(b). The *ε*_2_(*ω*) plot shows minor peaks at 4.53 eV, 4.72 eV and 4.66 eV for Na_3_InBr_6,_ K_3_InBr_6_ and Rb_3_InBr_6_, respectively. As the energy increases, major peaks of the imaginary part appear in the UV region at 6.27 eV, 6.24 eV and 6.16 eV, whereas the maximum peaks are observed at 9.78 eV, 8.50 eV and 8.34 eV for Na_3_InBr_6,_ K_3_InBr_6_ and Rb_3_InBr_6_, respectively. This trend suggests that the weak interband electronic transitions near the band edge cause the low-intensity peaks, but the intense UV peaks are caused by high-energy valence-to-conduction-band transitions. The shift and lowering of the maximum peak energies from Na to Rb imply increased polarizability and systematic alteration of the electronic structure *via* A-site substitution. Overall, the optical absorption of these halides is dominated by UV excitations, confirming their visual transparency. The refractive index defines the material's ability to slow down the speed of light as it moves through it.^70^ The refractive index of the studied halides is plotted in Fig. 6(c). The *n*(*ω*) at zero frequency is determined as 1.54, 1.47 and 1.46 for Na_3_InBr_6,_ K_3_InBr_6_ and Rb_3_InBr_6_, whereas significant peaks are observed in the UV region at 4.06 eV, 4.55 eV and 4.50 eV, respectively. The maximum peaks of *n*(*ω*) for Na_3_InBr_6,_ K_3_InBr_6_ and Rb_3_InBr_6_ are observed at 6.08 eV, 6.13 eV and 6.02 eV, respectively. The decrease in the static refractive index from Na_3_InBr_6_ to Rb_3_InBr_6_ indicates reduced electronic polarizability, which aligns with the growing bandgap trend. The pronounced UV peaks are caused by strong interband electronic transitions that promote light–matter interaction at higher photon energy. As a result, these halides show minimal dispersion in the visible and strong optical activity in the UV region. The extinction coefficient of optical materials is directly correlated with the imaginary part of the complex refractive index and indicates the loss of light intensity as a result of absorption within the medium. The extinction coefficient for the studied halides is presented in Fig. 6(d), where the minor extinction peaks for Na_3_InBr_6,_ K_3_InBr_6_ and Rb_3_InBr_6_ are observed at 4.58 eV, 4.74 eV and 4.66 eV, respectively. As the energy increases, significant peaks appeared in the UV region at 6.35 eV, 6.29 eV and 6.21 eV, respectively. The maximum peaks of the extinction coefficient for Na_3_InBr_6,_ K_3_InBr_6_ and Rb_3_InBr_6_ are observed at 12.23 eV, 9.75 eV and 12.80 eV, respectively. This suggests that mild optical losses occur near the band-edge as a result of tiny interband transitions, whereas substantial absorption and attenuation dominate in the UV region due to high electrical excitation. Thus, Na_3_InBr_6,_ K_3_InBr_6_ and Rb_3_InBr_6_ have little extinction in the visible region, indicating their applicability for UV optoelectronic applications. The optical reflectivity of a material is often defined as its potential to reflect incident light. The reflectivity plot for the studied halides is shown in Fig. 6(e). The static value of the reflectivity for Na_3_InBr_6,_ K_3_InBr_6_ and Rb_3_InBr_6_ is observed at 0.046, 0.037 and 0.035, respectively, whereas as the energy of the major peaks increased for Na_3_InBr_6_, occurring at 4.20 eV, 6.29 eV and 12.50 eV. For K_3_InBr_6_, prominent peaks are observed at 4.58 eV and 6.27 eV, whereas for Rb_3_InBr_6_, prominent peaks are observed at 4.50 eV and 6.16 eV. For all studied halides, the maximum reflectivity peaks are observed at 13.56 eV. This suggests that low static reflectivity confirms little light reflection in the low-energy region, implying high optical transparency. The optical conductivity measures a material's capacity to conduct electricity in response to an incident electromagnetic field at optical frequencies. It is derived from electronic interband and intraband transitions and represents frequency-dependent charge-carrier dynamics. The optical conductivity plots for Na_3_InBr_6,_ K_3_InBr_6_ and Rb_3_InBr_6_ are demonstrated in Fig. 6(f). The optical conductivity plots show minor peaks for Na_3_InBr_6,_ K_3_InBr_6_ and Rb_3_InBr_6_ at 4.58 eV, 4.72 eV and 4.66 eV, whereas strong peaks are observed at 6.27 eV, 6.24 eV and 6.16 eV, respectively. The maximum peaks of the optical conductivity for Na_3_InBr_6,_ K_3_InBr_6_ and Rb_3_InBr_6_ are observed at 9.83 eV, 9.70 eV and 8.39 eV, respectively. The absorption coefficient demonstrates a material's ability to absorb electromagnetic radiation per unit path length in the material. The absorption coefficient for Na_3_InBr_6_, K_3_InBr_6_ and Rb_3_InBr_6_ is plotted in Fig. 6(g). Minor peaks of the absorption coefficient for Na_3_InBr_6,_ K_3_InBr_6_ and Rb_3_InBr_6_ are observed at 4.55 eV, 4.69 eV and 4.72 eV, whereas prominent peaks are observed at 12.20 eV, 9.78 eV and 9.37 eV, respectively, in the UV region. The maximum absorption coefficient peaks for Na_3_InBr_6,_ K_3_InBr_6_ and Rb_3_InBr_6_ are observed at 13.56 eV, 12.99 eV and 12.93 eV, respectively. The energy loss refers to the amount of energy dissipated by fast-moving electrons as they go through a material due to inelastic scattering processes. The energy loss plot for Na_3_InBr_6,_ K_3_InBr_6_ and Rb_3_InBr_6_ is presented in Fig. 6(h). Minor energy loss peaks are observed at 4.72 eV, 4.80 eV and 4.72 eV, whereas prominent peaks appear at 6.21 eV, 6.38 eV and 6.40 eV, respectively. The maximum *L*(*ω*) for Na_3_InBr_6_, K_3_InBr_6_ and Rb_3_InBr_6_ is observed at 13.04 eV, 12.20 eV and 10.70 eV, respectively. This indicates that the low-energy loss features are caused by mild interband electronic excitations, but the pronounced peaks are caused by valence electron collective plasma oscillations. The shift in the highest loss peak from Na_3_InBr_6_ to Rb_3_InBr_6_ suggests a change in the plasma frequency and electron density due to A-site cation replacement. It is noted that the optical spectra indicate that A-site cation substitution (Na → K → Rb) primarily tunes the orbital hybridization indirectly through lattice expansion and structural distortion. Because the A-site cations (Na, K, Rb) have negligible direct contribution near the band-edges, their increasing ionic size weakens the In–Br orbital overlap by enlarging the bond lengths. This reduced hybridization leads to slight bandgap modification and a shift in the optical transitions, which is reflected in the graphs as peak shifts toward lower energies (red-shift) in the dielectric function, absorption coefficient, and energy loss spectra. Consequently, the refractive index and reflectivity also decrease slightly with heavier cations, confirming that A-site tuning controls the optical response *via* structural modulation rather than direct electronic contribution.

### Thermoelectric properties

**Fig. 7:**
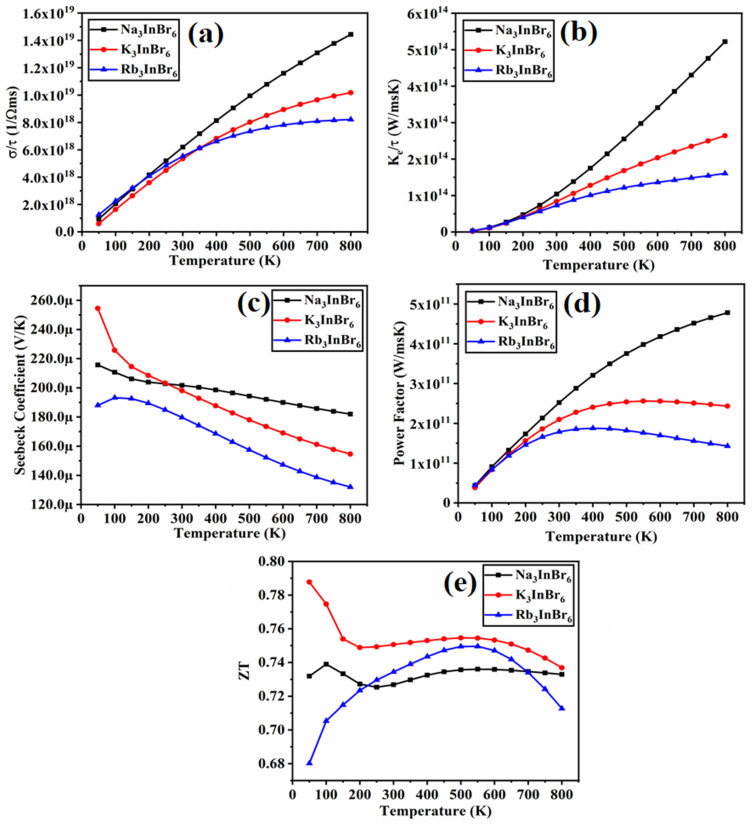
Thermoelectric properties of (Na/K/Rb)_3_InBr_6_.

Thermoelectric materials have been increasingly explored for the past few decades for their energy conversion applications. Thermoelectric materials possess the ability to directly transform the thermal power obtained from sun to electrical power.^71,72^ Utilizing the most efficient techniques to harvest electricity from the least expensive sources has been discovered to be very important by material scientists. Therefore, it becomes vital to clarify the operation of devices in order to explain their fundamental qualities and practical position. Waste heat and solar energy collection have enormous potential.^73^ Researchers are now considering materials that can effectively convert waste energy into green energy. Perovskite materials with their versatile crystal uniqueness and tunable properties have emerged as promising candidates for thermoelectric devices. Their flexibility allows for compositional engineering that can aid in optimizing the thermoelectric parameters, which are key parameters in assessing the thermoelectric efficiency. In this context, halide-based perovskite possess higher thermal stability, which makes them suitable high-performance thermoelectric devices.^73^ The sun is one of the main renewable energy sources. However, recent research has demonstrated that combining organic and inorganic components can result in novel halide perovskites.^74^ The effectiveness of thermoelectric materials is assessed based on several thermoelectric properties, which are computed with the help of semiclassical Boltzmann theory implemented in the BoltzTraP code.^75^ The thermoelectric properties of Na_3_InBr_6_, K_3_InBr_6_ and Rb_3_InBr_6_ are presented in Fig. 7. The electrical conductivity describes a material's ability to transfer charge carriers under an applied electric field. Materials with narrow bandgaps possess higher electrical conductivity and insulators or wide-bandgap semiconductors possess lower electrical conductivity. For the studied halides, the electrical conductivity shows an increasing trend as the temperature is increased, and the maximum values for Na_3_InBr_6,_ K_3_InBr_6_ and Rb_3_InBr_6_ are obtained as 1.44 × 10^19^ 1 Ω^−1^ m^−1^, 1.02 × 10^19^ 1 Ω^−1^ m^−1^ and 8.21 × 10^18^ 1 Ω^−1^ m^−1^ at 800 K, respectively, as depicted in Fig. 7(a). These results demonstrate that Na_3_InBr_6_ has a larger effective charge-carrier concentration, as compared to K_3_InBr_6_ and Rb_3_InBr_6_, suggesting more efficient charge transport at elevated temperatures. The thermal conductivity is also an important factor in analyzing the materials' thermoelectric performance. The thermal conductivity of the semiconducting materials comprises the lattice and electronic thermal conductivity, whereas the code we have employed only determines the electronic thermal conductivity.^76^ The thermal conductivity of the studied halides follows an increasing trend as the temperature is increased, and the maximum values obtained at 800 K are 55.21 × 10^14^ W m^−1^ s^−1^ K^−1^, 2.64 × 10^14^ W m^−1^ s^−1^ K^−1^ and 1.60 × 10^14^ W m^−1^ s^−1^ K^−1^ for Na_3_InBr_6,_ K_3_InBr_6_ and Rb_3_InBr_6_, respectively, as shown in Fig. 7(b). This implies that the studied halides become more efficient in conducting heat at higher temperatures, with Na_3_InBr_6_ exhibiting the highest thermal conductivity among the series. The trend also suggests that the A-site cation influences heat transport, decreasing from Na to Rb due to the increasing lattice size and associated phonon scattering. The Seebeck coefficient for Na_3_InBr_6,_ K_3_InBr_6_ and Rb_3_InBr_6_ is plotted in Fig. 7(c). The Seebeck coefficient demonstrates a material's ability to generate voltage in the presence of a temperature gradient.^77^ A higher Seebeck coefficient suggests that the material possesses higher capability for converting waste heat into electricity. The Seebeck coefficient has a maximum value of 215.67 µ(V K^−1^) at 50 K for Na_3_InBr_6_, which declines to 181.98 µ(V K^−1^) at 800 K. For K_3_InBr_6_, the maximum value is observed at 50 K as 215.67 µ(V K^−1^), which declines to 154.61 µ(V K^−1^) at 800 K. For Rb_3_InBr_6_, the maximum Seebeck coefficient is observed at 193.17 µ(V K^−1^) at 100 K, which declines to 131.96 µ(V K^−1^) at 800 K, as demonstrated in Fig. 7(c). All studied halides exhibit high thermoelectric efficiency at 50 K, whereas with a significant increase in temperature, the Seebeck coefficient declines. The Seebeck coefficient further demonstrates that Na_3_InBr_6_ is an effective material for converting waste heat to energy as compared to K_3_InBr_6_ and Rb_3_InBr_6_. The power factor indicates a material's efficiency to convert waste heat into electricity.^78^ The power factor's maximum value for Na_3_InBr_6_ is obtained as 4.78 × 10^11^ W m^−1^ s^−1^ K^−1^ at 800 K, whereas for K_3_InBr_6_, the maximum value is observed at 600 K as 2.55 × 10^11^ W m^−1^ s^−1^ K^−1^, which slightly declines to 2.43 × 10^11^ W m^−1^ s^−1^ K^−1^ when the temperature is raised to 800 K. For Rb_3_InBr_6_, the maximum value of the power factor is observed at 400 K as 1.87 × 10^11^ W m^−1^ s^−1^ K^−1^, which declines to 1.43 × 10^11^ W m^−1^ s^−1^ K^−1^ when the temperature is raised to 800 K, as shown in Fig. 7(d). The figure of merit is the most important parameter and plays a crucial role in assessment of the thermoelectric performance.^79^ High values of *ZT* indicate whether the material has high thermoelectric efficiency. Among the studied halides, the maximum value of *ZT* for Na_3_InBr_6_ is observed as 0.738 at 100 K, which declines to 0.72 at 250 K, and then increases to 0.732 when the temperature is raised to 800 K. The maximum value of *ZT* for K_3_InBr_6_ is obtained as 0.78 at 50 K, which drops to 0.74 at 200 K; when the temperature is increased, the *ZT* value remains steady till 600 K and a slight decline is observed in the value to 0.73 at 800 K. The *ZT* of Rb_3_InBr_6_ follows a parabolic path as the temperature is raised, and the maximum *ZT* is reported as 0.74 K at 550 K, which declines to 0.71 when the temperature is raised to 800 K, as illustrated in Fig. 7(e). The obtained values of *ZT* suggest that K_3_InBr_6_ and Na_3_InBr_6_ are viable for converting waste energy at elevated temperatures, whereas Rb_3_InBr_6_ is beneficial at moderate temperatures.

**Fig. 8:**
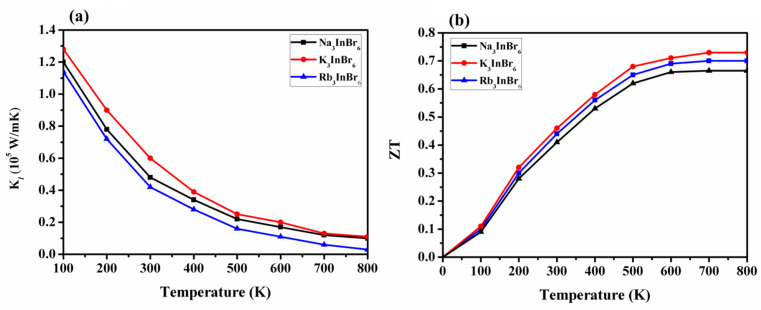
(a) Lattice thermal conductivity and (b) *ZT* plots of Na_3_InBr_6_, K_3_InBr_6_ and Rb_3_InBr_6_.

Using Slack's equation, the lattice thermal conductivity (*κ*_l_) was calculated, as illustrated in Fig. 8(a), and is given by the following expression:6
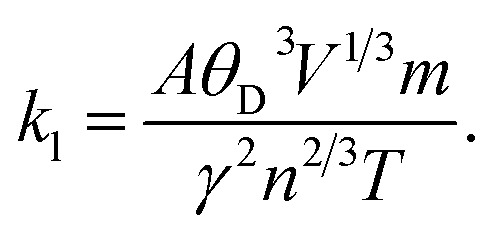


A represents a collection of physical constants (*A* ≈ 3.1 × 10^−8^), *γ* is the Grüneisen parameter, *θ*_D_ denotes the Debye temperature, and *V* corresponds to the atomic volume. The phonon vibrations exhibit a rapid increase, followed by a sharp decline over the temperature range of 100–800 K, suggesting an inverse correlation with the electronic contribution to the thermal conductivity for all materials. We investigated the influence of lattice vibrations on the *ZT* values to enhance their accuracy (Fig. 8(b)). The *ZT* values show a steady increase as the temperature rises. At 800 K, a *ZT* of 0.65, 0.7 and 0.73 is obtained for Na_3_InBr_6_, RB_3_InBr_6_ and K_3_InBr_6_, respectively. It can be observed that the inclusion of lattice vibrations has only a minimal effect on the *ZT* values across all the studied materials.

## Conclusion

This paper presents a first principles analysis of perovskite halides (Na/K/Rb)_3_InBr_6_ by employing the mBJ approximation and FP-LAPW approach. The negative ground state and formation energies indicate complete structural and thermodynamic stability. The studied halides exhibit cubic formation with minimum distortions, as predicted *via* the tolerance factor and octahedral tilting. The elastic constants computed using the stress-energy tensor matrix exhibits a decreasing trend with the replacement the A-site cation from Na to Rb. The material exhibits ductile behavior, which declines gradually when the A-site cation is replaced in order of Na → K → Rb. The elastic anisotropy estimated using the ELATE software shows that among the examined compounds, K_3_InBr_6_ has a lower anisotropy and a more uniform elastic response, whereas Na_3_InBr_6_ and Rb_3_InBr_6_ have significant directional shifts. The electronic properties indicate direct bandgaps of 3.37 eV, 3.84 eV, and 3.86 eV for Na_3_InBr_6_, K_3_InBr_6_ and Rb_3_InBr_6_, respectively. The optical properties of Na_3_InBr_6,_ K_3_InBr_6_ and Rb_3_InBr_6_ showed strong dispersion and polarization peaks in the UV region, making these materials potential candidates for ultraviolet optoelectronic applications, such as UV photonic devices. Furthermore, the thermoelectric properties of Na_3_InBr_6,_ K_3_InBr_6_ and Rb_3_InBr_6_ indicate that these halides are excellent candidates for the conversion of waste energy into renewable energy.

## Conflicts of interest

The authors have no conflict of interest.

## Data Availability

Data will be available from the authors upon reasonable request.
